# Two Sides of the Same Coin: ERP and Wavelet Analyses of Visual Potentials Evoked and Induced by Task-Relevant Faces

**DOI:** 10.5709/acp-0195-3

**Published:** 2016-12-31

**Authors:** Rob H. J. Van der Lubbe, Izabela Szumska, Małgorzata Fajkowska

**Affiliations:** 1 Cognitive Psychology, University of Finance and Management, Warsaw, Poland; 2 Cognitive Psychology and Ergonomics, University of Twente, The Netherlands; 3 Institute of Psychology, Polish Academy of Science, Warsaw, Poland

**Keywords:** EEG, ERPs, Wavelet analyses, Evoked power, Induced power, Phase Reset model, Evoked model

## Abstract

New analysis techniques of the electroencephalogram (EEG) such as wavelet analysis open the possibility to address questions that may largely improve our understanding of the EEG and clarify its relation with related potentials (ER Ps). Three issues were addressed. 1) To what extent can early ERERP components be described as transient evoked oscillations in specific frequency bands? 2) Total EEG power (TP) after a stimulus consists of pre-stimulus baseline power (BP), evoked power (EP), and induced power (IP), but what are their respective contributions? 3) The Phase Reset model proposes that BP predicts EP, while the evoked model holds that BP is unrelated to EP; which model is the most valid one? EEG results on NoGo trials for 123 individuals that took part in an experiment with emotional facial expressions were examined by computing ERPs and by performing wavelet analyses on the raw EEG and on ER Ps. After performing several multiple regression analyses, we obtained the following answers. First, the P1, N1, and P2 components can by and large be described as transient oscillations in the α and θ bands. Secondly, it appears possible to estimate the separate contributions of EP, BP, and IP to TP, and importantly, the contribution of IP is mostly larger than that of EP. Finally, no strong support was obtained for either the Phase Reset or the Evoked model. Recent models are discussed that may better explain the relation between raw EEG and ERPs.

## Introduction

In the last decades, the number of studies in the neighboring fields of cognitive neuroscience, electrophysiology, biological psychology, and cognitive psychology employing measures derived from the electroencephalogram (EEG) has increased enormously. Often, EEG is used to improve our understanding of the underlying processes triggered by the presentation of a stimulus or the emission of a response, but EEG is also measured to determine the specific state of an individual (e.g., whether someone is awake and highly vigilant or drowsy).

 The standard approach to study event-related processes (e.g., related to a stimulus or a response), introduced by Dawson (1951), is to average activity time-locked to certain events belonging to the same category, thereby creating so-called event related potentials (ERPs). The idea is that the raw EEG contains both relevant activity, related to a specific event, and irrelevant activity that actually may be a combination of noise and unrelated background EEG activity. The relevant signal is thought to be time-locked to a certain event and to be small in magnitude, whereas the irrelevant activity is temporally unrelated to this event and is thought to be very large. After averaging across a substantial number of artefact-free trials this may leave the relevant event-related (or evoked) activity (e.g., see Handy, 2005; Heinze, Münte, & Mangun, 1994; Luck, 2005) while the irrelevant activity cancels out. This view on the origin of ERPs is generally known as the Evoked model (Jervis, Nichols, Johnson, Allen, & Hudson, 1983; Lopes da Silva, 1999). Despite the clear resulting peaks and troughs after the averaging procedure, which have been related to processes like stimulus encoding, stimulus discrimination, attentional reorienting, conflict monitoring, motor activation, and error processing, the exact origin of the ERP is not that clear (e.g., see Sauseng et al., 2007; and see below). 

 An alternative class of approaches to study stimulus-related processes, denoted as time-frequency analyses, is currently gaining in popularity (e.g., see Cohen, 2014; Gross, 2014). One such method, which will be focused upon here, is wavelet analysis. The general idea of time-frequency analyses is that not all relevant EEG activity is strictly phase-locked (or evoked) to the event of interest (e.g., see Buszáki, 2006). For example, Berger (1929) already revealed that the ongoing raw EEG changes upon stimulus presentation, as the alpha rhythm (~8-12 Hz) strongly reduces after a stimulus and is often replaced by the more high-frequent beta rhythm (~13-20 Hz). Obviously, this activity shortly before stimulus onset is mostly not visible in ERPs due to cancellation, nevertheless, this pre-stimulus baseline activity may have a crucial impact on the observed ERPs (e.g., see Fellinger, Klimesch, Gruber, Freunberger, & Doppelmayr, 2011; Gruber et al., 2014; Klimesch, 2011). Time-frequency analyses enable us to determine the presence of oscillatory patterns in different frequency bands over time. Thus, with wavelet analyses, it can be established whether oscillatory activity in a specific frequency band, often expressed in power (squared amplitude), increases or decreases relative to a certain event. 

 The goal of the current paper is to increase our understanding of early stimulus-related EEG activity by combining both approaches in different ways. Three closely related issues will be addressed. The first issue concerns the question to what extent observed ERP components can be understood as the summation of transient evoked oscillations in specific frequency bands. For example, can the early visual P1 and N1 components (e.g., see Klimesch, 2011; Klimesch et al., 2004) be understood as the peak and trough of an evoked (or Phase Reset, see below) alpha oscillation (i.e., alpha-ringing), or are these two ERP components related to different frequency bands? The second issue relates to the question whether the ERP approach may not lead to a serious underestimation of stimulus-related activity (e.g., see Makeig, Debener, Onton, & Delorme, 2004). By performing wavelet analyses on both the raw EEG and ERPs, we may be able to determine the contribution of evoked (stimulus-locked) and induced activity (not phase-locked but stimulus-related activity) to overall observed activity, and therefore may assess whether a significant amount of information is lost. A third issue concerns the origin of the ERP. If ERP components can be described as a summation of transient evoked oscillations in various frequency bands, is it then possibly the case that these evoked oscillations are related to pre-stimulus baseline activity? For example, a prominent model (i.e., the Phase Reset model) states that ERPs originate from a phase-reset of ongoing oscillations ( Başar, 1999; see already Savers, Beagley, & Henshall, 1974). If this view is correct then one might argue that ERPs are more indicative of the state of an individual rather than that they reflect different processing stages of these stimuli. The general approach in this paper is to address the aforementioned issues by employing multiple linear regressions, which will allow us to determine the presence and the strength of the relation between the different relevant variables. 

### ERP Components Described as the Sum of Evoked Oscillations in Specific Frequency Bands

The first issue may be rephrased as the extent to which the amplitude of the ERP within a specific time window Δ*t* can be described as a linear combination of the evoked power (EP) in different frequency bands (*f*_i_; *i* = 1 to *n*) in this same time window. This relation is indicated in equation 1. The contribution of a frequency band to the ERP is indicated by the regression coefficient (*c*_i_; *i* = 1 to *n*).

*ERP*Δ*t* = Σ_i_ *c*_i_ *EP*(*f*_i_)Δ*t* (1)

 After computing ERPs for a large number of individuals, wavelet analyses can be performed on individual ERPs. Subsequently, the amplitude of the individual ERPs within a specific time window can be determined together with the power in different frequency bands in this same time window. Next, the relation between the dependent (left side of Equation 1) and independent variables or predictors (right side of Equation 1) can be estimated by performing a multiple linear regression analysis. The following seven frequency bands were selected: lower theta (θ_1_), middle theta (θ_2_), upper theta (θ_3_), lower alpha (α_1_), upper alpha (α_2_), lower beta (β_1_), and upper beta (β_2_). An implication of the choice for these frequency bands is that a part of the ERP may be left unexplained. This contribution, which is most likely related to power in the delta (δ) band, may be estimated by including a constant in the regression analyses. As individual differences in estimated power may be very large and its distribution will be skewed, we computed the logarithm (log_10_) of the estimated power values and used the transformed data for the regression analyses (e.g., see Koopman, Wouters, & Krijzer, 1996). In these specific regression analyses, we employed a step-in step-out approach, which implies that only those predictors are included that show a significant contribution. 

### Total Power Decomposed in Evoked, Baseline, and Induced Power

 The second issue concerns the extent to which overall stimulus-related activity is due to evoked and induced activity. If the contribution of induced activity is very large, then focusing solely on ERPs as an index of stimulus-related processing is maybe too limited (e.g., see Buszáki, 2006; Makeig et al., 2004) as a substantial part of relevant activity is ignored. Here, we adopted an approach related to the research by Hanslmayr et al. (2007). The idea is that overall activity or total power (TP) within a specific time window Δ*t*, which can be separated in different frequency bands (*f*_i_; *i* = 1 to *n*), is a linear function of evoked power (EP), power in the pre-stimulus baseline (BP), and induced power (IP) related to the onset of the stimulus. 

*TP*(*f*_i_)Δ*t* = *a*_i_ *EP*(*f*_i_)Δ*t* + *b*_i_ *BP*(*f*_i_)+ *IP*(*f*_i_)Δ*t* (2)

The regression coefficients *a*_i_ and *b*_i_ specify the contribution of EP and BP to TP. For each individual, wavelet analyses can be performed on single trials. Subsequently, the average power per frequency band can be computed for a specific time window Δ*t*, which specifies TP(*f*_i_)_Δ*t*_. The same approach already described above can be applied to ERPs, which computes EP(*f*_i__)Δ*t*_. Furthermore, power in the pre-stimulus baseline can be determined by selecting a constant time interval before stimulus presentation BP(*f*_i_), which is identical to TP(*f*_i_)_Δ*t*_ when Δ*t* concerns a time interval before stimulus onset. The term that cannot directly be derived from the data is induced power, IP(*f*_i_)_Δ*t*_. However, TP is by definition a combination of EP, BP, and IP, as power is either directly related to a stimulus (EP), or it is not directly related (IP and/or BP). Thus, IP is in principle that part of the data (TP) that cannot be explained by EP and BP (i.e., the intercept). Again, the relation between the relevant variables can be estimated by performing a multiple linear regression analysis. By including a constant term in the regression analyses, the part of the TP that cannot be explained by EP and BP can be determined, which provides an estimate of IP.

### The Relation Between Pre-Stimulus Baseline Power and Evoked Power

 The third issue to be addressed concerns the relation between pre-stimulus oscillations (i.e., pre-stimulus baseline activity) and evoked oscillatory activity, which may describe (see issue 1) the different exogenous ERP components. Multiple studies have tried to test the Phase Reset model (Başar, 1999; Savers et al., 1974). This model states that ERPs are actually the result of a phase reset of ongoing pre-stimulus oscillations at stimulus onset while the overall power in various frequency bands is thought to remain the same (e.g., see Makeig et al., 2004 , 2002). Several ideas have been proposed that may account for the presence of a phase reset at stimulus onset. For example, Burgess (2012) proposed the Firefly model of synchronization, which relates phase synchronization in specific frequency bands to an increase in neuronal communication triggered by an internal or external event. Klimesch, Sauseng, Hanslmayr, Gruber, and Freunberger (2007) proposed that stimuli may induce an event-related phase reorganization (ERPR) that plays a crucial role in the timing of neural processes. An alternative view, also pointed out by Başar (1999) , may be based on the idea that the onset of an event induces a resonance of ongoing oscillations, which may actually depend on their phase at stimulus onset. The major alternative to the Phase Reset model, the Evoked model, holds that ERPs are evoked responses generated by a specific event that are superimposed on and independent of ongoing oscillations (e.g., see Mäkinen, Tiitinen, & May, 2005). Sauseng and colleagues (2007) evaluated the presented arguments and the up to that moment reported empirical support for the Phase Reset and the Evoked model, and concluded that no unambiguous support has been presented for either of the two models. One of the evaluated arguments concerned the relation between pre-stimulus oscillations and observed ERP components: If the Phase Reset model is correct, then the power of pre-stimulus oscillations should influence the ERP as there is only a reset of the phase of ongoing oscillations. It was argued that testing this aspect may be problematic due to too low activity and due to filtering artefacts that originate from procedures like wavelet analysis. Although there may indeed be a problem with filtering artefacts when oscillations are examined at stimulus onset, this influence seems small when activity is determined relatively early within the pre-stimulus baseline. Furthermore, nearly all studies tried to address this issue by focusing on the raw EEG, which implies a very low signal-to-noise ratio and thus the possibility that noise biases the outcome. However, a relation between pre-stimulus oscillations and ERP components should also show up in individual differences, which is the approach taken here. 

To address the third issue, we used a comparable approach as before. Now, the idea is that EP for a specific frequency band *f*_i_ within a specific time window Δ*t* may be a linear function of induced power in the pre-stimulus baseline (BP) and a constant CP(*f*_i_)Δ*t*, which explains changes in EP that cannot be described by BP.

EP(*f*_i_)Δ*t* = *d*_i_ BP(*f*_i_) + CP(*f*_i_)Δ*t* (3)

The regression coefficient *d*_i_ describes the extent to which BP predicts EP. The relation between the variables can again be determined by performing a multiple linear regression analysis and including a constant. If the data reveal that the relation between EP and BP is very small or even absent while there is a clear contribution of CP, then the data seem in line with the Evoked model. However, a very strong relation between EP and BP and a small contribution of CP would support the idea that ongoing oscillations largely determine evoked responses, in line with the Phase Reset model. In that case, the model employed for the comparison between EP and TP (Equation 2) has to be reconsidered as EP and BP can no longer be considered as unrelated components that simply add up.

## Methods

### Participants

One hundred and thirty participants took part in the experiment, who were recruited from the student population of several universities in Warsaw. All participants had normal or corrected-to-normal visual acuity and had no history of neurological diseases. Due to procedural and some technical errors, the EEG data of seven participants could not be used, which left 123 participants for the analyses (100 females, 23 males; *M*_age_ = 25.0, *SD* = 7.0, ranging from 19 to 50 years; five left-handed). A local ethics committee approved the experimental procedures. The experiment was conducted with the informed and written consent of each participant. Participants took part in two sessions on two separate days. In the first session, several questionnaires had to be filled in, not detailed further, and in the second session (one week later) an emotional go/nogo task had to be carried out while the EEG was being measured.

### Stimuli and Procedure

The set of stimuli consisted of 240 of Ekman and Friesen’s (1976) colored pictures of emotional facial expressions, which included angry, sad, happy, and neutral faces. Pictures were taken from 27 individuals (18 males).

Each trial started with a grey background (38 cd/m²) being presented for 1,500 ms. Subsequently, the relevant stimulus (a picture with a specific emotional expression) was presented in the center. Stimulus presentation ended after a response was given or after 500 ms, whichever came first. The size of all stimuli was 9.3º × 11.7º.

In three different blocks, participants were asked to press a response key with their preferred index finger as quickly and accurately as possible when they detected the go stimulus. The go stimulus varied per block, being either an angry, happy, or sad face. Before the start of each block, participants were informed which category required a response. The three blocks of 240 trials each contained 120 go trials (angry-go; happy-go; sad-go) and 120 nogo trials composed of 40 neutral trials and two sets of 40 angry, happy, or sad trials depending on the type of block. In the following, these blocks are indicated as the Angry, Happy, and Sad blocks, which refers to the stimulus that required a response. The order of the trials was randomized and the order of the blocks was counterbalanced. The duration of the task was approximately 25 min.

### Apparatus and EEG Recordings

Participants were seated in a darkened room at approximately 70 cm in front of a 22-inch LED monitor with a refresh rate of 60 Hz. Stimuli were presented by using Presentation software (Neurobehavioral System, Inc.). EEG was recorded from 32 active electrodes attached to an electrode cap (ActiCap, BrainProducts GmbH) located at standard 10-20 system positions, which was referenced on-line to an electrode located at FCz. Facial EMG (not reported) was recorded as well. EEG was registered with BrainVision Recorder (BrainProducts GmbH) installed on a separate computer. Signals were sampled at 1,000 Hz per channel and were amplified with a 72-channels DC amplifier (QuickAmp, BrainProducts GmbH). A high cutoff filter of 100 Hz and a notch filter of 50 Hz were used. The impedance was kept below 5 kΩ for all EEG electrodes.

### Data Analysis

Our interest here concerned the EEG data. Therefore, we did not focus on the acquired behavioral data.

#### EEG data

EEG was analyzed using Brain Vision Analyzer (Version 2.0.1.3931; Brain Products GmbH, Munich, Germany). The raw EEG data were first filtered by a Butterworth Zero Phase filter with a low cutoff of 0.16 Hz, and a high cutoff of 40 Hz. Next, ICA (Independent Component Analysis) was employed to extract signals that were considered as having a non-cortical origin (e.g., being due to blinks, saccades, muscle activity, and heart rate). Subsequently, a time window was selected from -800 until 1,850 ms relative to the markers that signaled the presentation of the facial expressions. A baseline correction of -100 to 0 ms was applied. Trials with artefacts (gradient criterion: 50 μV/ms; min-max criterion: -/+ 150 μV; low activity criterion: 0.5 μV per 100 ms) were removed. Next, nogo trials for each block were selected for the EEG analyses, which we chose to avoid possible interference from movement-related activity. Next, two different EEG analyses were performed for the nogo trials in the three separate blocks (Happy, Angry, Sad).

**Figure 1.**
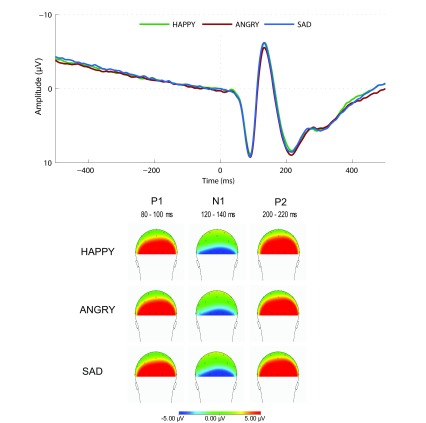
The grand average ER Ps at the occipital electrode O2 for the nogo stimuli in the Happy, Angry, and Sad blocks. The topographical maps of the P1, N1, and P2 components clearly display an occipital focus, which justifies the decision to focus on the O2 electrode in our analyses

 In the first analysis, we initially determined the nogo ERPs per individual by averaging across all nogo trials, separately per block. Subsequently, a wavelet analysis (a complex Morlet with Gabor normalization, *c* = 5) was applied on the individual ERPs, which allows us to estimate EP in different frequency bands related to the nogo stimuli. We extracted the power (in μV^2^) of seven frequency bands, starting from the lower theta to the upper beta band, which were separated in seven logarithmic steps (see also Van der Lubbe, Bundt, & Abrahamse, 2014; Van der Lubbe & Utzerath, 2013). The following bands were specified: θ_1_ (3.2-4.8 Hz; Gaussian lower and upper band, respectively; lower theta), θ_2_ (4.2-6.3 Hz; middle theta), θ_3_ (5.5-8.2 Hz; upper theta), α_1_ (7.2-10.7 Hz; lower alpha), α_2_ (9.4-14.0 Hz; upper alpha), β_1_ (12.2-18.4 Hz; lower beta), and β_2_ (16.0-24.0 Hz; upper beta). Next, we determined the averaged EP per band in 20 ms time windows from 40 to 300 ms after stimulus onset on the O2 electrode. This electrode was selected as the P1, N1, and P2 components were all clearly visible at this site in the three blocks (see Figure 1). Individual ERP amplitudes were also assessed for the same 20 ms time windows on the O2 electrode. The relation between ERP amplitude and obtained power in the different frequency bands was subsequently assessed by performing a stepwise multiple linear regression analysis for several 20 ms time window for the three blocks (see below). 

In the second analysis, we performed wavelet analyses with the same parameter settings as above but now directly on the raw EEG. Thus, analyses were performed on the same trials but before averaging them. This implies that we included both evoked and induced power. Next, an average was created, which can be considered as an estimate of TP. Subsequently, the averaged TP for each band was determined for the aforementioned 20 ms time windows, all with the same parameter settings as in the other analyses. Additionally, we estimated BP in the various frequency bands for the interval from -500 to -200 ms relative to stimulus onset.

The logarithm (log_10_) was computed for all obtained power values. These transformed values were used for all statistical analyses that were performed with IBM SPSS statistics (version 23).

#### Multiple Linear Regression

To estimate to what extent the proposed relations in equations 1, 2, and 3 hold, three different multiple linear regression models were tested for the nogo stimuli in all three blocks. In the first regression model (related to Equation 1), the amplitude of the ERP within a specific 20-ms time window at O2 was the dependent variable, and EP in the seven frequency bands for the same time window plus a constant were used as the predictor variables. In this case, a stepwise multiple linear regression analysis was carried out. To minimize statistical Type I errors, the critical *p*_in_-value was set at 0.05/(number of predictors). The number of predictors amounted to 8 (constant, plus the coefficients for the θ_1_ up to the β_2_ bands). The exclusion criterion pout was set at 2 × *p*_in_, which is the standard setting for this criterion (*p*_in_ <= 0.00625, *p*_out_ > 0.0125). The regression analyses were performed for those intervals in which clear ERP components were observed.

 In the second regression model (related to Equation 2), TP within a specific time window was the dependent variable, and EP, BP, and a constant (an estimate of the remaining IP) were used as the predictor variables. As we examined 13 time windows, seven frequency bands, and included three predictors, we had to minimize Type I errors. The criterion of significance for at least two successive time windows was employed (see Van der Lubbe et al., 2014). This implies that the critical p value for two successive time windows amounted to 0.014 (=√[0.05/12×7×3]). 

In the third regression model (related to Equation 3), EP within a specific time window was used as the dependent variable, while BP and a constant were used as predictor variables. As we examined seven frequency bands and included two predictor variables, we now employed a significance criterion of 0.004 (=0.05/[7×2]).

## Results

### EEG Data

**Figure 2.**
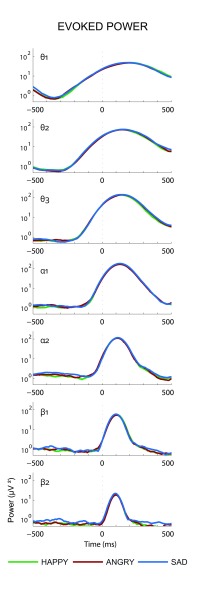
The grand average after logarithmic transformation of individually estimated evoked power (in μV^2^) at the occipital electrode O2 for the lower theta (θ_1_) up to the higher beta (β_2_) bands determined on the basis of the ERPs based on nogo stimuli in the Happy, Angry, and Sad blocks. Values along the Y-axis concern a logarithmic scale.

**Figure 3.**
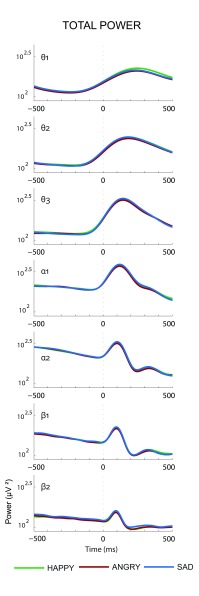
The grand average after logarithmic transformation of estimated total power (in μV^2^) at the occipital electrode O2 for the lower theta (θ_1_) up to the higher beta (β_2_) bands determined on the basis of the raw EEG on nogo stimuli in the Happy, Angry, and Sad blocks. Values along the *Y*-axis concern a logarithmic scale.

Grand average ERPs for the nogo stimuli in the three blocks for the right occipital site O2 are displayed in Figure 1. Topographies of the P1, N1, and P2 components in the three blocks are also displayed. Grand averages of the results of the wavelet analyses performed on the individual ERPs for the seven frequency bands, which estimate EP on O2 for the three blocks, are presented in Figure 2. Furthermore, the grand average results of the wavelet analyses on the raw EEG, which estimate TP, are displayed in Figure 3.

### Issue 1: ERP Components Described as the Sum of Evoked Oscillations in Specific Frequency Bands

The P1, N1, and P2 components reached largest amplitudes in the 80-100 ms, the 120-140 ms, and the 200-220 ms time windows, respectively. Our goal was to assess what frequency bands strongly contribute to the ERP components in this study. Therefore, we restricted our analyses to these three time windows.

#### The 80-100 ms interval (the P1 component)

The analyses for the Happy block showed a highly significant relation between three predictors (θ_1_, α_2_, and a constant; *R*^2^ = .41) and P1 amplitude, *F*(2, 120) = 41.4, *p* < .001. Inspection of the regression coefficients (see Table 1) indicates that EP in the α_2_ band had a positive contribution to P1 amplitude, while EP in the θ_1_ band and the constant had a dampening effect. Analyses for the angry nogo stimuli revealed a nearly identical pattern. A significant relation was observed between three predictors (θ_1_, α_2_, and a constant; *R*^2^ = .61) and P1 amplitude, *F*(2, 120) = 93.2, *p* < .001. The same pattern was observed for the sad nogo stimuli as a significant relation was observed between three predictors (θ_1_, α_2_, and a constant; *R*^2^ = .52) and P1 amplitude, *F*(2, 120) = 63.9, *p* < .001. In all cases, EP in the α_2_ band had a positive contribution while EP in the θ_1_ band and the constant had a dampening effect on P1 amplitude.

**Table 1.** Mean Event-Related Potential (ERP) Amplitudes on Nogo Trials in the Happy, Angry and Sad Blocks

Block		Happy		Angry		Sad	
window	variable	EP (s.e.)	*c*_i_ (s.e.)	EP (s.e.)	*c*_i_ (s.e.)	EP (s.e.)	*c*_i_ (s.e.)
80-100 ms	ERP	8.3 (0.6)		8.6 (0.6)		8.6 (0.6)	
P1	constant		-5.9* (2.0)		-6.3** (1.5)		-5.7* (1.9)
	θ_1_	1.57 (0.06)	-2.7** (0.8)	1.55 (0.06)	-3.7** (0.6)	1.60 (0.05)	-5.3** (0.9)
	θ_2_	1.82 (0.05)		1.81 (0.05)		1.82 (0.06)	
	θ_3_	2.07 (0.05)		2.05 (0.05)		2.10 (0.05)	
	α_1_	2.15 (0.04)		2.13 (0.04)		2.18 (0.04)	
	α_2_	2.02 (0.04)	9.2** (1.0)	2.00 (0.04)	10.3** (0.8)	2.02 (0.05)	11.2** (1.0)
	β_1_	1.74 (0.04)		1.70 (0.05)		1.76 (0.04)	
	β_2_	1.29 (0.05)		1.24 (0.05)		1.34 (0.05)	
120-140 ms	ERP	-5.4 (0.7)		-4.8 (0.6)		-5.6 (0.7)	
N1	constant		9.7** (2.0)		9.6** (1.8)		10.7** (1.9)
	θ_1_	1.62 (0.06)		1.61 (0.06)		1.65 (0.05)	-4.9* (1.6)
	θ_2_	1.87 (0.05)	-8.1** (1.0)	1.86 (0.05)	-7.8** (0.9)	1.87 (0.05)	-4.4* (1.5)
	θ_3_	2.13 (0.05)		2.13 (0.05)		2.15 (0.05)	
	α_1_	2.22 (0.04)		2.20 (0.04)		2.24 (0.04)	
	α_2_	2.04 (0.04)		2.01 (0.04)		2.04 (0.04)	
	β_1_	1.68 (0.04)		1.64 (0.05)		1.65 (0.05)	
	β_2_	1.09 (0.05)		1.06 (0.05)		1.10 (0.05)	
200-220 ms	ERP	8.1 (0.6)		8.7 (0.6)		8.4 (0.6)	
P2	constant		-3.3 (2.7)		-3.5 (2.7)		-3.4 (2.6)
	θ_1_	1.68 (0.05)		1.66 (0.05)		1.67 (0.05)	
	θ_2_	1.78 (0.06)		1.81 (0.05)		1.82 (0.05)	
	θ_3_	1.99 (0.05)		2.03 (0.04)	6.1** (1.3)	2.04 (0.04)	
	α_1_	1.94 (0.04)	5.9** (1.3)	1.91 (0.05)		1.97 (0.04)	6.0** (1.3)
	α_2_	1.33 (0.05)		1.30 (0.05)		1.36 (0.05)	
	β_1_	0.56 (0.04)		0.49 (0.05)		0.47 (0.06)	
	β_2_	0.01 (0.04)		0.01 (0.05)		0.04 (0.06)	

*Note*. *p* < 0.00625, ** *p* < 0.001. Mean event-related potential (ERP) amplitudes on nogo trials in the Happy, Angry and Sad blocks are indicated for the time windows in which the P1, N1, and P2 components were observed. Log10 transformed evoked power (EP) values are displayed for the lower theta (θ_1_), the middle theta (θ_2_), the upper theta (θ_3_), the lower alpha (α_1_), the upper alpha (α_2_), the lower beta (β_1_), and the upper beta (β_2_) bands. Relevant regression coefficients (*c*_i_) indicate the relation between the observed ERP component and EP in a specific frequency band. Standard errors (*SE*) are indicated between brackets.

#### The 120-140 ms interval (the N1 component)

The analyses for the happy nogo stimuli showed a highly significant relation between two predictors (θ_2_, and a constant; *R*^2^ = .35) and N1 amplitude, *F*(1, 121) = 65.2, *p* < .001. Inspection of the regression coefficients (see Table 1) indicates that EP in the θ_2_ band contributed to N1 amplitude, while the constant had a dampening effect. An almost identical pattern (see Table 1) was observed for the angry nogo stimuli (*R*^2^ = .38), *F*(1, 121) = 74.3, *p* <.001. For the sad nogo stimuli, the analyses showed a significant relation between three predictors (θ_1_, θ_2_, and a constant; *R*^2^ = .41) and N1 amplitude, *F*(2, 120) = 41.1, *p* < .001. Inspection of the regression coefficients indicates that EP in the θ_1_ and the θ_2_ band contributed to N1 amplitude, while the constant had a dampening effect.

#### The 200-220 ms interval (the P2 component)

The analyses for the happy nogo stimuli showed a significant positive relation between one predictor (α_1_; *R*^2^ = .14) and P2 amplitude, *F*(1, 121) = 19.8, *p* < .001. The analyses for the angry nogo stimuli showed that one predictor (θ_3_, *R*^2^ = .15) was related to P2 amplitude, *F*(1, 121) = 21.9, *p* < .001. For the sad nogo stimuli again only one predictor (α_1_; *R*^2^ = .15) showed a significant positive relation with P2 amplitude, *F*(1, 121) = 21.5, *p* < .001. Thus, the positive relation between P2 amplitude and EP (see Table 1) either concerns the lower α band or the highest θ band. The lower values for *R*^2^ indicate that the relation between EP and ERP amplitude became less strong.

### Issue 2: Total Power Decomposed in Evoked, Baseline, and Induced Power

**Figure 4.**
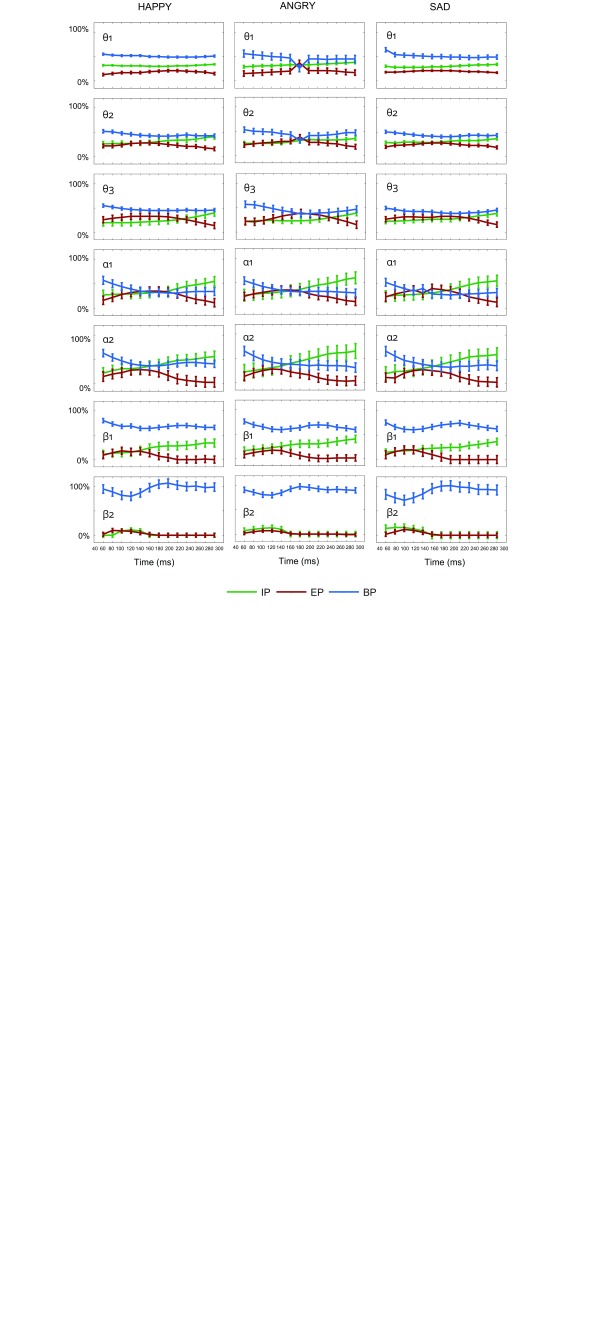
The estimated contribution of induced power (IP), evoked power (EP), and pre-stimulus baseline power (BP) in percentage of the total power (TP) at the occipital electrode O2 from 40 to 300 ms after stimulus onset.

Grand averages of the results of the wavelet analyses performed on the raw EEG for the seven frequency bands, which estimates TP on O2 for the three blocks, are presented in Figure 3. The decomposition of TP in EP, BP, and IP is displayed in Figure 4. Estimated percentages of BP and EP were determined by multiplying the obtained regression coefficients with the observed BP and EP and scaling this by TP. The remaining part to be explained is indicated by the constant, which provides an estimate for IP. Results are reported for each block. We excluded those components (EP, BP and IP) for which the regression coefficients did not satisfy our criterion value (*p* < .014 for at least two successive time windows). We report only those time windows for which no significant contribution was observed as the majority of the time windows met our criteria.

#### Happy nogo stimuli

For the θ_1_ band, explained variance (*R*^2^) by the predictors (IP, EP, BP) for the full time range from 40 to 300 ms was very high (from .71 to .85). On average, the estimated contribution of IP, EP, and BP amounted to 31%, 18% and 51% (see Figure 4). For the θ_2_ band, *R*^2^ varied from .74 to .87, and the contributions of IP, EP, and BP were estimated at 32%, 23%, and 45%. For the θ_3_ band, *R*^2^ varied from .72 to .88, and the average contributions of IP, EP, and BP were estimated at 26%, 28%, and 47%. For the α_1_ band, *R*^2^ varied from .59 to .86. Here, the contributions of IP, EP, and BP were estimated at 37%, 25%, and 37%. For the α_2_ band, *R*^2^ varied from .49 to .88, and the contributions of IP, EP, and BP were estimated at 40%, 16%, and 44%. For the β_1_ band, *R*^2^ varied from .60 to .92. Here, the average contributions of IP, EP, and BP were estimated at 24%, 8%, and 69%. From the 200-220 until the 280-300 ms window, the regression coefficient for EP no longer satisfied our significance criterion. For the β_2_ band, *R*^2^ varied from .80 to .91. Here, the average contributions of IP, EP, and BP were estimated at 2%, 3%, and 96%. IP did not significantly contribute until the 80-100 ms window and also not after the 120-140 ms time window. EP did no longer contribute to TP after the 140-160 ms time window.

#### Angry nogo stimuli

For the θ_1_ band, *R*^2^ varied from .80 to .85. On average, the estimated contribution of IP, EP, and BP amounted to 33%, 20%, and 46%. For the θ_2_ band, *R*^2^ varied from .77 to .88, and the contributions of IP, EP, and BP were estimated at 30%, 26%, and 45%. For the θ_3_ band, *R*^2^ varied from .73 to .90, and the contributions of IP, EP, and BP were estimated at 27%, 28%, and 45%. For the α_1_ band, *R*^2^ varied from .57 to .91. Here, the contributions of IP, EP, and BP were estimated at 39%, 25%, and 36%. For the α_2_ band, *R*^2^ varied from .42 to .88. The contributions of IP, EP, and BP were estimated at 44%, 15%, and 41%. For the β_1_ band, *R*^2^ varied from .56 to .88. Here, the contributions of IP, EP, and BP were estimated at 28%, 7%, and 65%. For the 200-220 and the 220-240 ms time windows, the regression coefficient for EP was no longer significant. For the β_2_ band, *R*^2^ varied from .80 to .92. Here, the contributions of IP, EP, and BP were estimated at 4%, 2%, and 90%. From the 140-160 ms time window, IP gave no significant contribution, while EP did not contribute to TP for the 160-180 and the 180-200 ms time windows.

#### Sad nogo stimuli

For the θ_1_ band, *R*^2^ varied from .76 to .84. The average contributions of IP, EP, and BP amounted to 30%, 19%, and 51%. For the θ_2_ band, *R*^2^ varied from .78 to .87, and on average the contributions of IP, EP, and BP were estimated at 31%, 24%, and 44%. For the θ_3_ band, *R*^2^ varied from .72 to .89, and the average contributions of IP, EP, and BP were estimated at 28%, 29%, and 43%. For the α_1_ band, *R*^2^ varied from .60 to .92. Here, the contributions of IP, EP, and BP were estimated at 37%, 28%, and 35%. For the α_2_ band, *R*^2^ varied from .44 to .88, and the contributions of IP, EP, and BP were estimated at 41%, 16%, and 42%. For the β_1_ band, *R*^2^ varied from .59 to .88. Here, the contributions of IP, EP, and BP were estimated at 24%, 7%, and 68%. The regression coefficient for EP was not significant from the 180-200 till the 280-300 ms time window. For the β_2_ band, *R*^2^ varied from .83 to .91. Here, the contributions of IP, EP, and BP were estimated at 5%, 3%, and 90%. IP no longer contributed after the 120-140 ms time window, while EP played no longer a significant role after the 140-160 ms time window.

### Issue 3: The Relation Between Pre-Stimulus Baseline Power and Evoked Power

The goal of these analyses was to determine whether EP in the different frequency bands, which may account for the P1, N1, and P2 components, was strongly related to pre-stimulus baseline activity (BP). As we were interested in the time intervals where the P1, N1, and P2 showed their maxima, we restricted our analyses to the 80-100 ms, the 120-140 ms, and the 200-220 ms time windows.

#### The 80-100 ms interval (the P1 component)

For the happy nogo stimuli, BP in the α_2_, β_1_, and β_2_ bands was a positive predictor of EP in the respective frequency bands (see Table 2). Most relevant seems BP in the α_2_ band as this significantly predicted the P1 component. Thus, these data seem in line with the Phase Reset model. However, we also observed that CP in the θ_3_, α_1_, and α_2_ band was a significant predictor, which indicates that individual differences in these frequency bands have an additional source, favoring the Evoked model. For the angry nogo stimuli (see Table 3), BP proved to be a significant predictor of EP for the θ_3_ up to the β_1_ band, but we also observed that CP in both α bands was a positive predictor. For the sad nogo stimuli, BP proved to be a significant predictor of EP for the θ_3_ up to the β_2_ band, but CP in the α_1_ band was also a positive predictor. Together, these findings seem to support both the Phase Reset and the Evoked model. Importantly, however, explained variance remains quite low, indicating that BP and CP are no strong predictors of EP.

**Table 2.** The Outcome of Multiple Regression Analyses for Nogo Stimuli in the Block With Happy Go Stimuli for the Lower Theta (θ_1_) up to the Higher Beta Band (β_2_)

Happy													
		80-100 ms				120-140 ms				200-220 ms			
Band	BP	EP	*d*_i_	CP	*R*^2^	EP	*d*_i_	CP	*R*^2^	EP	*d*_i_	CP	*R*^2^
θ_1_	2.08 (0.02)	1.57(0.06)	0.38(0.26)	0.77(0.55)	.02	1.62 (0.06)	0.25(0.27)	1.10(0.56)	.01	1.68 (0.05)	0.18(0.24)	1.31(0.50)	.01
θ_2_	2.06 (0.02)	1.82 (0.05)	0.39(0.22)	1.01(0.44)	.03	1.87 (0.05)	0.41(0.21)	1.02(0.43)	.03	1.78 (0.06)	0.45(0.23)	0.85(0.48)	.03
θ_3_	2.09 (0.02)	2.07 (0.05)	0.37(0.17)	1.30**(0.37)	.04	2.13 (0.05)	0.38(0.18)	1.33**(0.37)	.04	1.99 (0.05)	0.45(0.18)	1.05(0.37)	.05
α_1_	2.32 (0.04)	2.15 (0.04)	0.25(0.11)	1.57**(0.26)	.04	2.22 (0.04)	0.26(0.10)	1.62**(0.24)	.05	1.94 (0.04)	0.29(0.10)	1.26**(0.24)	.06
α_2_	2.42 (0.03)	2.02 (0.04)	0.47**(0.11)	0.89*(0.28)	.12	2.04 (0.04)	0.39**(0.10)	1.10**(0.25)	.11	1.34(0.05)	0.25(0.12)	0.73(0.30)	.03
β_1_	2.24 (0.02)	1.74 (0.05)	0.67**(0.16)	0.24(0.36)	.13	1.68 (0.04)	0.66**(0.16)	0.20(0.37)	.12	0.56 (0.05)	0.66**(0.18)	-0.93(0.41)	.10
β_2_	2.12 (0.02)	1.29 (0.05)	0.66**(0.19)	-0.12(0.40)	.09	1.09 (0.05)	1.10**(0.21)	-1.24(0.45)	.18	0.01 (0.04)	0.64**(0.18)	-1.36**(0.38)	.10

*Note*. *p* < 0.004, ** *p* < 0.001. Prestimulus baseline power (BP) was used as predictor for the observed evoked power (EP) within a specific time window that corresponds either with the P1, the N1, or the P2 component. Regression coefficients for each frequency band are indicated with *d*_i_. We also determined the possible contribution of a constant, indicated with CP. The total amount of explained variance by the analysis is indicated with *R*^2^. All power values were log10 transformed before performing the analyses. Standard errors (SE) are indicated between brackets.

**Table 3.** The Outcome of Multiple Regression Analyses for Nogo Stimuli in the Block With Angry Go Stimuli for the Lower Theta (θ_1_) up to the Higher Beta Band (β_2_)

Angry													
		80-100 ms				120-140 ms				200-220 ms			
Band	BP	EP	*d*_i_	CP	*R*^2^	EP	*d*_i_	CP	*R*^2^	EP	*d*_i_	CP	*R*^2^
θ_1_	2.07 (0.02)	1.55 (0.06)	0.55 (0.24)	0.42 (0.51)	.04	1.61 (0.06)	0.55 (0.24)	0.48 (0.50)	.04	1.66 (0.05)	0.52 (0.23)	0.59 (0.47)	.04
θ_2_	2.05 (0.02)	1.81 (0.05)	0.59 (0.21)	0.60 (0.42)	.06	1.86 (0.05)	0.58 (0.21)	0.67 (0.42)	.06	1.81 (0.05)	0.60 (0.21)	0.58 (0.44)	.06
θ_3_	2.09 (0.02)	2.05 (0.05)	0.58* (0.20)	0.84 (0.41)	.07	2.13 (0.05)	0.61** (0.17)	0.86 (0.36)	.10	2.03 (0.04)	0.62** (0.15)	0.73 (0.32)	.12
α_1_	2.32 (0.04)	2.13 (0.04)	0.36* (0.11)	1.31** (0.26)	.08	2.20 (0.04)	0.35* (0.11)	1.40** (0.25)	.08	1.91 (0.05)	0.34 (0.12)	1.14** (0.28)	.06
α_2_	2.43 (0.03)	2.00 (0.04)	0.45** (0.11)	0.91** (0.27)	.12	2.01 (0.04)	0.41** (0.11)	1.03** (0.27)	.10	1.30 (0.05)	0.29 (0.13)	0.60 (0.32)	.04
β_1_	2.24 (0.02)	1.70 (0.05)	0.69** (0.18)	0.14 (0.42)	.11	1.64 (0.05)	0.76** (0.17)	-0.06 (0.39)	.14	0.49 (0.05)	0.64* (0.20)	-0.93 (0.46)	.08
β_2_	2.12 (0.02)	1.24 (0.05)	0.56 (0.21)	0.07 (0.45)	.05	1.06 (0.05)	0.62* (0.20)	-0.25 (0.44)	.07	0.01 (0.05)	1.04** (0.21)	-2.21** (0.44)	.18

*Note*. *p* < 0.004, ** *p* < 0.001. Prestimulus baseline power (BP) was used as predictor for the observed evoked power (EP) within a specific time window that corresponds either with the P1, the N1, or the P2 component. Regression coefficients for each frequency band are indicated with *d*_i_. We also determined the possible contribution of a constant, indicated with CP. The total amount of explained variance by the analysis is indicated with *R*^2^. All power values were log10 transformed before performing the analyses. Standard errors (*SE*) are indicated between brackets.

#### The 120-140 ms interval (the N1 component)

For the happy nogo stimuli, significant BP predictors for EP were found in the α_2_ and both β bands but not in the most relevant θ_2_ band (see Table 1). The contribution of CP was significant in the θ_3_ and both α bands. For the angry nogo stimuli, significant BP predictors were found in the θ_3_ up to the β_2_ bands, but here we also observed significant contributions of CP in both α bands. For the sad nogo stimuli, we observed significant BP predictors from the α_1_ up to the β_2_ band, and we observed significant contributions of CP in both α bands. Again, these findings seem to provide some support for the Phase Reset but also for the Evoked model. However, BP in the θ_2_ band was not identified as a relevant predictor, even though it was shown to be the main predictor of the N1 component, and the explained variance remains low.

#### The 200-220 ms interval (the P2 component)

Significant BP predictors for EP for the happy nogo stimuli were present in both β bands. However, we also observed that CP in the α_1_ band was a significant positive predictor, which is the band that seems most relevant for the P2 component (see Table 1). For the angry nogo stimuli, significant BP predictors were found in the θ_3_ and both β bands. CP in the α_1_ band was again a significant positive predictor. Finally, for the sad nogo stimuli significant positive BP predictors were present in the θ_3_ up to the β_2_ band, while CP in the α_1_ band was a significant positive predictor. In most cases, explained variance remains low. The major exception here is CP in the β_2_ band, which in all conditions was a negative predictor for EP. At the same time BP in the β_2_ band was a positive predictor. Inspection of Figures 2 and 4 reveals that EP in this time window is absent (the regression coefficients for EP were also non-significant or just significant). Therefore, a relation of EP with BP and CP seems not very meaningful. As BP has a strong positive value, CP will simply be estimated to be negative to end up with a value around zero.

## Discussion

Three issues were raised in our Introduction that will be addressed by considering the outcome of three different multiple regression analyses. The first issue concerns the extent to which ERP components can be described as the sum of transient evoked oscillations in different frequency bands. The second issue deals with the extent to which overall EEG activity in a specific frequency band (TP) after presenting a visual stimulus (i.e., here facial stimuli that require no response) can be ascribed to stimulus-locked activity (EP), stimulus-induced but not time-locked activity (IP), and pre-stimulus baseline activity (BP). The third issue concerns the extent to which stimulus-locked activity (EP) is due to pre-stimulus baseline activity (BP).

### ERP Components Described as the Sum of Evoked Oscillations in Specific Frequency Bands

 We focused on three successive ERP components (P1, N1, and P2) that all had a clear occipital focus. With regard to the P1 component, the results of the three different blocks (see Table 1) clearly revealed that its amplitude is strongly positively related to EP in the α_2_ band, while a negative relation was observed with EP in the θ_1_ band. Additionally, we observed significant contributions of a constant in all blocks. This also concerned a negative relation with P1 amplitude, and might imply contribution of the lower δ band. Thus, it seems that activity in the θ (and possibly δ) band(s) has a dampening effect on P1 amplitude while activity in the α_2_ band boosts the P1 component. The relation between α power and the P1 component might underlie the same functionality (see Klimesch, 2011; Klimesch et al., 2004). As activity in the α band is generally interpreted as inhibition, one might argue that the P1 component also reflects inhibition (i.e., the P1 inhibition timing hypothesis proposed by Klimesch, 2011). Such a view implies that the interpretation of earlier results like the enhancement of the P1 component due to visual attention (e.g., see Van der Lubbe & Woestenburg, 1997) requires an update, as this effect is unlikely to reflect gain modulation. However, one could argue that a transient α oscillation like the P1 component is not necessarily related to more tonic oscillations in the α band (see Mishra, Martínez, Schroeder, & Hillyard, 2012). Nevertheless, several studies (see also below) indicate that evoked α power is related to pre-stimulus baseline α power (e.g., see Fellinger et al., 2011; Gruber et al., 2014; Himmelstoss et al., 2015; see also below). 

 The results of our analyses for the N1 time window revealed quite consistent results in all three blocks (see Table 1). Namely, the N1 component showed strong negative relations with EP in the θ_2_ and also once the θ_1_ band. At the same time, positive relations were observed with the constants in the different blocks. These findings suggest that N1 amplitude is boosted by EP in the θ band (see also Klimesch et al., 2004), while it is also dampened by power in the δ band. Although frontal θ has been related to focal attention (Onton, Delorme, & Makeig, 2005), more widespread and also posterior θ is generally linked with drowsiness and deactivation. Therefore, an interpretation of the posterior N1 component as the reflection of a stimulus discrimination process (Vogel & Luck, 2000) may also become a point of discussion (see also Van der Lubbe, Vogel, & Postma, 2005). Again, a transient θ oscillation cannot directly be equated with more tonic oscillations in the θ band, although a clear relation between evoked θ power and pre-stimulus baseline θ power (but see below) would support a common underlying functionality. 

Our observations regarding the P2 time window revealed a slightly less consistent pattern (see Table 1). For two blocks, P2 amplitude had a strong positive relation with EP in the α_1_ band, while in the Angry block, there was a positive relation with EP in the θ_3_ band. These different results might point to subtle differences between this block and the other blocks. Most importantly, the results again show that effects mainly concern frequency bands that are related with inhibition or deactivation.

Altogether, the outcome of these analyses suggests that early ERP components can very well be described as reflecting the sum of EP in various frequency bands. Furthermore, the P1 and N1 components seem related to different oscillatory frequencies and do not reflect alpha ringing. Most importantly, the strong relation between different ERP components and specific oscillations may imply that generally accepted interpretations of effects on these components need to be reconsidered.

### Total Power Decomposed in Evoked, Baseline, and Induced Power

 Several authors argued that it may very well be the case that most stimulus-related activity is not strictly time-locked to stimulus onset, which implies that focusing on ERPs may be too limited as a major part of relevant activity is averaged out ( Buszáki, 2006; Makeig et al., 2004). The current analyses allow us to determine the validity of this idea, by assessing the separate contributions of IP, EP, and BP to TP. Estimated contributions from 40 to 300 ms after stimulus onset for the different frequency bands and three blocks are displayed in Figure 4. The patterns observed in the different blocks are quite comparable, though not identical. 

For the θ_1_ band the estimated contributions of IP, EP, and BP, averaged across blocks and all time windows amounted to 31%, 19%, 49%. Obviously, the contribution of EP is small, while the contribution of BP is remarkably high. Importantly, IP clearly adds to TP, which implies that a major part of stimulus-related but not phase-locked activity is removed after performing the averaging procedure. For the θ_2_ band, the contributions of IP, EP, and BP are estimated at 31%, 24%, 44%, and for the θ_3_ band, the contributions of IP, EP, and BP are estimated at 27%, 28%, 44%. Thus, the pattern in the different θ bands is more or less the same.

For the α_1_ band the estimated contributions of IP, EP, and BP, averaged across blocks and all time windows amounted to 38%, 26%, and 36%. For the α_2_ band, the contributions of IP, EP, and BP are estimated at 42%, 16%, and 42%. Thus, the contribution of IP for the α bands is even higher than for the θ bands, while the contribution of EP becomes smaller. Furthermore, the contribution of BP remains strong. Inspection of Figure 4 also reveals that in general the contribution of IP to TP increases over time, while the contribution of EP decreases.

For the β_1_ band, the estimated contributions of IP, EP, and BP amounted to 25%, 8%, and 67%, and for the β_2_ band, the estimated contributions were 6%, 2%, and 92%. Here strong reductions were observed for IP and especially for EP, while TP seems largely due to BP (see Figure 4).

Together, our results support the aforementioned idea that focusing solely on ERPs, thus evoked activity, ignores a major part of stimulus-related activity. This especially concerns activity in the θ and α bands, while activity in the β_2_ appears not stimulus-related. The latter observation does not exclude the possibility that this frequency band plays another role by transmitting information by phase changes, but apparently these phase changes are not related to stimulus onset, otherwise they should have become visible in EP. A more general observation is the strong presence of BP for all frequency bands. This suggests that a major part of EEG activity after stimulus onset is not phase reset by this stimulus. Furthermore, the major presence of induced activity in most frequency bands also indicates that activity that is not phase-locked cannot be considered as irrelevant background activity. The latter two observations seem quite relevant for the discussion of our third issue.

### The Relation Between Pre-Stimulus Baseline Power and Evoked Power

 Are ERPs indeed due to a phase-reset of ongoing oscillations, as was proposed by Başar (1999) and several other researchers (e.g., Makeig et al., 2002), or can they be interpreted as unique stimulus-evoked neural responses? We showed when considering our first issue that early ERP components can be largely described as the sum of EP in various frequency bands. Therefore, this question may be rephrased as whether and to what extent EP at a specific moment after stimulus onset is strongly related to BP. In our introduction, we indicated that a strong relation between BP and EP would support the Phase Reset model, while a strong contribution of CP to EP would rather be in line with the Evoked model. 

If we focus on the 80-100 ms time window and the most relevant frequency band for the P1 component (the α_2_ band, see Table 1), then it seems that in the Happy block (see Table 2) BP predicts EP ([0.47 × 2.42]/2.02 = 56%), while the contribution of CP (44%) remains substantial. In the Angry block (see Table 3), BP in the α_2_ band predicts EP (54%), while CP again has a strong contribution (46%). Finally, in the Sad block (see Table 4), BP predicts EP (64%), while CP has a clear contribution (36%). Thus, evidence is obtained for both the Phase Reset and the Evoked model. Importantly, explained variance (see Tables 2 to 4) remains rather low (.12 to .16), which indicates that a substantial amount of variance in EP is not explained.

**Table 4.** The Outcome of Multiple Regression Analyses for Nogo Stimuli in the Block With Happy Go Stimuli for the Lower Theta (θ_1_) up to the Higher Beta Band (β_2_)

Sad													
		80-100 ms				120-140 ms				200-220 ms			
Band	BP	EP	*d*_i_	CP	*R*^2^	EP	*d*_i_	CP	*R*^2^	EP	*d*_i_	CP	*R*^2^
θ_1_	2.09 (0.02)	1.60 (0.05)	0.29 (0.22)	1.00 (0.47)	.01	1.65 (0.05)	0.23 (0.22)	1.17 (0.47)	.01	1.67 (0.05)	0.20 (0.23)	1.25 (0.48)	.01
θ_2_	2.05 (0.02)	1.82 (0.06)	0.65 (0.23)	0.49 (0.46)	.06	1.87 (0.05)	0.60 (0.22)	.64 (0.45)	.06	1.82 (0.05)	0.55 (0.22)	.69 (0.45)	.05
θ_3_	2.08 (0.02)	2.10 (0.05)	0.52* (0.17)	1.02 (0.36)	.07	2.15 (0.05)	0.50 (0.18)	1.10 (0.38)	.06	2.04 (0.04)	0.55** (0.16)	0.89 (0.33)	.09
α_1_	2.31 (0.04)	2.18 (0.04)	0.43** (0.10)	1.17** (0.24)	.13	2.24 (0.04)	0.40** (0.10)	1.31** (0.23)	.13	1.97 (0.04)	0.37** (0.10)	1.10** (0.24)	.10
α_2_	2.43 (0.03)	2.02 (0.05)	0.53** (0.11)	0.73 (0.27)	.16	2.04 (0.04)	0.48** (0.10)	0.88** (0.25)	.16	1.36 (0.05)	0.39** (0.10)	0.41 (0.28)	.09
β_1_	2.25 (0.02)	1.76 (0.04)	0.79** (0.15)	-0.02 (0.34)	.19	1.65 (0.05)	0.82** (0.17)	-0.19 (0.39)	.16	0.47 (0.06)	0.81** (0.20)	-1.35* (0.46)	.12
β_2_	2.14 (0.02)	1.34 (0.05)	0.75** (0.17)	-0.27 (0.37)	.13	1.10 (0.05)	0.72** (0.20)	-0.44 (0.43)	.10	0.04 (0.06)	1.40** (0.19)	-2.95** (0.42)	.30

Note. *p* ˂ 0.004, ** *p* ˂ 0.001. Prestimulus baseline power (BP) was used as predictor for the observed evoked power (EP) within a specific time window that corresponds either with the P1, the N1, or the P2 component. Regression coefficients for each frequency band are indicated with *d*_i_. We also determined the possible contribution of a constant, indicated with CP. The total amount of explained variance by the analysis is indicated with *R*^2^. All power values were log_10_ transformed before performing the analyses. Standard errors (*SE*) are indicated between brackets.

With regard to the 120-140 ms time window and the most relevant frequency band for the N1 component (the θ_2_ band), we observed no significant predictors for EP in any of the blocks. Furthermore, explained variance was very low (.03 to .06), indicating that most of the variance of EP remains unexplained. Significant predictors for EP were identified in the higher θ_3_ up to β_2_ bands, both in BP and CP, but as already indicated these bands had no major contribution to the N1 component.

The P2 component was observed in the 200-220 ms time window. The most relevant frequency band is either the α_1_ band (for the Happy and Sad block), or the θ_3_ band (for the Angry block). For the Happy block, BP in the α_1_ band showed no significant contribution, while CP had a strong contribution (65%). For the Angry block, BP in the θ_3_ band predicted EP (64%), while CP showed no significant contribution. Finally, for the Sad block, BP in the α_1_ band predicted EP (43%), while CP had an even stronger contribution (56%). Explained variance for the relevant frequency bands varied from .06 to .12, which once again shows that an important part of the data is left unexplained.

Together, it seems that there is no strong evidence for either the Phase Reset or the Evoked model. Furthermore, we should mention that a small relation between BP and EP may be due to individual differences in thickness of the skull and the folding pattern of the cortex, as they will affect both. This might explain the relation that we observed here. Other aspects of our data seem also difficult to reconcile with the Phase Reset model. A strong version of this model would predict that BP after stimulus onset is replaced by EP. Inspection of Figure 4, however, shows that BP remains quite high, while the small contribution of EP never clearly exceeds BP. At the same time, the Phase Reset model cannot explain the clear presence of IP. However, the Evoked model faces the same problem as non-evoked activity is considered to be background activity. In the following section we will examine whether some recently proposed models may provide a better explanation for our data than the Evoked and the Phase Reset model.

### Alternative Models Accounting for the Role of Pre-Stimulus Baseline Power on Evoked Activity

 Our focus was restricted to early ERP components and we concluded that the Phase Reset model provides only a minor contribution to EP. Other studies clarified that the Phase Reset model cannot account for later (often denoted as endogenous) ERP components (e.g., see Barry, 2009). However, the Evoked model seemed also insufficient. Thus, the relation between pre-stimulus baseline activity and early ERP components appears to be more intricate than a simple phase reset. In the last decade, several studies revealed that spatial attention modulates baseline activity in especially the α band (e.g., see Thut, Nietzel, Brandt, & Pascual-Leone, 2006; Van der Lubbe & Utzerath, 2013; Worden, Foxe, Wang, & Simpson, 2000). The general observation is that when attention is directed at one side of the visual field, α power is larger at ipsilateral than at contralateral occipital sites, and this increased ipsilateral α power is thought to reflect inhibition. Recent ideas became even more specific (Jensen & Mazaheri, 2010; Mathewson, Gratton, Fabiani, Beck, & Ro, 2009; Mazaheri & Jensen, 2008 ; Schalk, 2015). Schalk (2015) proposed the function-through-biased-oscillations (FBO) hypothesis, which states that oscillatory voltage rather than oscillatory power reflects cortical excitability. This may show up in more efficient behavior and increased amplitudes of early ERP components (see Mathewson et al., 2009). In the case of strong oscillations in the α band, there are transient phases of facilitation and inhibition, which depend on the phase (peak or trough) of the oscillation, while in the case of weak oscillations, there is a more tonic phase of facilitation. This view implies a more indirect relation between pre-stimulus baseline activity and evoked (but also induced power), which may explain the low amount of explained variance of EP. Furthermore, this hypothesis also implies cross-frequency coupling between BP and EP as a reduction in α oscillations may release a slow oscillation like the contingent negative variation (CNV). An appropriate test of this model seems to require the analysis and selection of single trials, which is beyond the scope of the current approach. 

 Klimesch et al. (2007) presented an alternative idea. They proposed the Event-Related Phase Reorganization (the ERPR) model, which implies that only those oscillations that are task relevant will be reset, while other oscillations remain unaffected. This idea may explain why in our analyses only weak support was obtained for a phase reset, as we focused only on the NoGo stimuli that required no response, although they were intermixed with Go stimuli. A proper test of this model might be a comparison of different tasks with identical stimuli, requiring either simple stimulus detection or stimulus discrimination. 

### Some Caveats of the Chosen Analyses

The choice of certain parameter settings in our analyses may have some limitations. First, we employed a log10 transformation of the individually obtained power values (in μV2). This approach was chosen to reduce major individual differences. However, a disadvantage of this transformation is that the observed relation between the different variables is not so easy to interpret. Another, probably more insightful approach is to perform the analyses with non-squared amplitudes1 and leave out the transformation. Secondly, the chosen value of the *c* parameter for the wavelet analyses (5) implies that the temporal resolution is not the highest. This results in a smoothing of the estimated power, which is well visible in Figure 2, as the evoked power in the α and θ bands starts before stimulus onset. Nevertheless, as a logarithmic scale was employed this effect looks worse than it really is. We determined BP in the 500-200 ms window, which implies that for most of our results this filtering artefact played no role. Nevertheless, it may have some influence for the θ_1_ and θ_2_ bands. An implication may be that the contribution of BP to TP is overestimated for these sub-bands. Furthermore, it might explain part of the correlation between BP and EP in these frequency bands. However, inspection of Tables 2 to 4 shows no such effect, which indicates that the chosen parameter value induced no major problem. The advantage of a low temporal resolution is a better frequency resolution, which also implies that individual differences in peak latency of EP will play no major role. Nevertheless, one might consider the possibility to lower the c parameter to 3, which will improve the temporal resolution.

## Conclusions

In conclusion, the P1, N1, and P2 components can by and large be described as transient oscillations in the α and θ bands. The contribution of IP to TP is mostly larger than of EP, which confirms that focusing on EP ignores an important part of stimulus-related activity. Finally, no strong support was obtained for either the Phase Reset or the Evoked model, which implies that new views on the origin of ERPs have to be developed and tested.
